# The 2021 FASEB science research conference on NAD metabolism and signaling

**DOI:** 10.18632/aging.203766

**Published:** 2021-12-09

**Authors:** Vera Gorbunova, Marcus Buschbeck, Xiaolu A. Cambronne, Karthikeyani Chellappa, Daniela Corda, Juan Du, Marc Freichel, Jonathan Gigas, Alexander E. Green, Feng Gu, Iva Guberovic, Aravinthkumar Jayabalan, Imrankhan Khansahib, Sarmistha Mukherjee, Andrei Seluanov, Matthew A. Simon, Lars J. Sverkeli, Nora Kory, Daniel C. Levine, Ivan Matic, Andrey Nikiforov, Johannes G.M. Rack, Shin-Ichiro Imai, David A. Sinclair, Debra Toiber, Yongjuan Zhao, Raul Mostoslavsky, Lee Kraus, Andreas H. Guse

**Affiliations:** 1 Departments of Biology and Medicine, University of Rochester, Rochester, NY 14627, USA; 2 Cancer and Leukaemia Epigenetics and Biology Program, Josep Carreras Leukaemia Research Institute (IJC), Campus ICO-GTP-UAB, Badalona, Catalonia 08916, Spain; 3 Program for Predictive and Personalized Medicine of Cancer, Germans Trias i Pujol Research Institute (PMPPC-IGTP), Badalona, Catalonia 08916, Spain; 4 Department of Molecular Biosciences, University of Texas at Austin, Austin, TX 78705, USA; 5 Department of Physiology and Institute for Diabetes, Obesity and Metabolism, Perelman School of Medicine, University of Pennsylvania, PA 19104, USA; 6 Department of Biomedical Sciences, National Research Council, Rome 00185, Italy; 7 Department of Structural Biology, Van Andel Institute, Grand Rapids, MI 49503, USA; 8 Institute of Pharmacology, Heidelberg University, Heidelberg, Baden-Württemberg 69117, Germany; 9 Department of Biology, University of Rochester, Rochester, NY 14627, USA; 10 Ottawa Institute of Systems Biology, Interdisciplinary School of Health Sciences, Faculty of Health Sciences, University of Ottawa, Ottawa, ON K1N 6N5, Canada; 11 Éric Poulin Centre for Neuromuscular Disease, Department of Biochemistry, Microbiology and Immunology, Faculty of Medicine, University of Ottawa, Ottawa, ON K1N 6N5, Canada; 12 The Calcium Signalling Group, Department of Biochemistry and Molecular Cell Biology, University Medical Center Hamburg-Eppendorf, Hamburg 20246, Germany; 13 Department of Biochemistry and Molecular Biology, Johns Hopkins Bloomberg School of Public Health, Baltimore, MD 21205, USA; 14 Department of Biological Sciences, University of Bergen, Bergen, Vestland 5007, Norway; 15 Department of Molecular Metabolism, Harvard T. H. Chan School of Public Health, Boston, MA 02115, USA; 16 Department of Medicine, Division of Endocrinology, Metabolism, and Molecular Medicine, Feinberg School of Medicine, Northwestern University, Chicago, IL 60611, USA; 17 Max Planck Institute for Biology of Ageing, Cologne, Nordrhein-Westfalen 50931, Germany; 18 Institute of Cytology, Russian Academy of Sciences, St. Petersburg 199178, Russia; 19 Sir William Dunn School of Pathology, University of Oxford, Oxford, Oxfordshire OX1 3RE, UK; 20 Department of Developmental Biology, Department of Medicine, Washington University School of Medicine, St. Louis, MO 63110, USA; 21 Department of Gerontology, Laboratory of Molecular Life Science, Institute of Biomedical Research and Innovation, Kobe, Hyogo 650-0047, Japan; 22 Genetics Department, Blavatnik Institute, Harvard Medical School, Boston, MA 02115, USA; 23 Department of Life Sciences, Ben-Gurion University of the Negev, Beer Sheva, 84105, Israel; 24 Ciechanover Institute of Precision and Regenerative Medicine, School of Life and Health Sciences, The Chinese University of Hong Kong, Shenzhen, Guangdong 518172, China; 25 Massachusetts General Hospital Cancer Center, Harvard Medical School, Boston, MA 02115, USA; 26 The Broad Institute of Harvard and MIT, Cambridge, MA 02114, USA; 27 Laboratory of Signaling and Gene Regulation, Cecil H. and Ida Green Center for Reproductive Biology Sciences, University of Texas Southwestern Medical Center, Dallas, TX 75390, USA; 28 Division of Basic Research, Department of Obstetrics and Gynecology, University of Texas Southwestern Medical Center, Dallas, TX 75390, USA

**Keywords:** NAD, metabolism, signaling, PARP, sirtuins, aging

## INTRODUCTION

Nicotinamide adenine dinucleotide (NAD+) serves as a signaling molecule or a co-enzyme in multiple critical biological reactions. The enzymes whose activities are dependent on NAD+ such as PARPs and Sirtuins, have a diverse biology in health and disease, and represent potential nutritional and therapeutic targets to prevent or treat aging, cancer, and metabolic dysregulation.

The conference “NAD+ Metabolism and Signaling” originally planned as a full in-person meeting was rescheduled for 2022 due to the pandemic. To give the researchers an opportunity to meet in 2021, the organizers decided to hold an online conference on June 15 and 16, 2021. The objective of the 2021 conference was to provide a platform for junior researchers to present their latest scientific results. Therefore, majority of the presentations were chosen from the submitted abstracts, with a limited number of invited keynote lectures. Conference’s topics were ‘NAD signaling’, ‘poly(ADP-ribosyl)ation and PARPs’, ‘sirtuins’, and ‘metabolism and interventions’. FASEB ‘NAD Metabolism and Signaling 2021’ is part of a series with previous conferences held in Dublin (2019), New Orleans (2017), Hamburg/Timmendorfer Strand (2015), Itasca/Chicago (2013), and Barga/Lucca (2011).

## NAD signaling

The first session on Tuesday, June 15, 2021, was devoted to NAD + signaling and chaired by Andreas Guse (Hamburg, Germany). Several endogenous derivatives of NAD are Ca^2+^mobilizing second messengers. Examples are cyclic ADP-ribose (cADPR) and nicotinic acid adenine dinucleotide phosphate (NAADP) that were discovered more than 25 years ago [1, 2], and ADP-ribose whose Ca^2+^ entry promoting role was described 20 years ago [3]. In the session, three talks covered NAADP signaling, while two talks reported new insights into the multifunctional enzyme SARM1. Finally, the (very) unusual structural feature of ion channel TRPM2 possessing two active ligand binding sites was highlighted.

In the first keynote Marc Freichel from Heidelberg University focused on a group of novel players involved in controlling or regulating Ca^2+^ release from acidic intracellular stores, termed ‘organellar Ca^2+^ regulators’ (OCaRs). In both pancreatic acinar cells and cardiomyocytes, Marc and his team identified OCaR1 as a novel gatekeeper of Ca^2+^ release from TPC2-containing and NAADP-sensitive acidic granules in acinar cells preventing uncontrolled exocytosis and OCaR2 as the central orchestrator of catecholamine-evoked Ca^2+^ release that originates from NAADP-sensitive acidic organelles.

In the first short presentation, Feng Gu (Hamburg, Germany) presented evidence for a new enzyme that forms NAADP under physiological conditions (pH value and membrane topology). In T cells, gene knock-out of the new candidate gene resulted in largely decreased initial, local Ca^2+^ signals, termed Ca^2+^ microdomains, while knock-out of *Cd38*, a gene for a multifunctional enzyme that *in vitro* can produce NAADP, was without effect.

A recently discovered receptor/binding protein for NAADP was presented by Imrankhan Khansahib (Hamburg, Germany). HN1L/JPT2 was purified by chromatographical separation and identified in an enriched fraction by mass spectrometry. Knock-out of the protein in human and rat T cells decreased initial Ca^2+^ microdomains and partially also global Ca^2+^ signaling. Co-localization studies using super-resolution microscopy and co-immunoprecipitation indicate interaction with ryanodine receptors as potential NAADP-sensitive Ca^2+^ channels [4].

The rodenticide vacor appears to be converted by SARM1 to a cytotoxic NAD analog in HEK293 cells, as detailed by Lars Sverkeli (Bergen, Norway) in short presentation #3. Accumulation of the cytotoxic NAD analog was accompanied by both NAD depletion and accumulation of cADPR. In cells lacking SARM1, a multifunctional enzyme similar in substrate- and reaction-specificity as CD38, cell viability was not affected, indicating that SARM1 enzyme activity is necessary for vacor toxicity.

In the fourth short presentation, Yongjuan Zhao (Shenzhen, China) reported the discovery of novel fluorescent probe and inhibitors of SARM1. Using the novel fluorescent probe, SARM1 enzymatic activity was imaged in live cells [5]. An analogue of nisoldipine was identified as covalent inhibitor of SARM1 reacting with cysteine residues. Excitingly, neurons were protected from axonal degeneration by this intervention, due to an inactive conformation of SARM1. With the probe, acidic pH was discovered to activate SARM1 and revealed the critical role of a salt bridge between ARM and TIR domains for maintaining the auto-inhibition [6].

In the early career keynote Juan Du (Grand Rapids, USA) reported recent advances of ligand recognition and gating mechanism of TRPM2, a non-specific cation channel gated by 2’deoxy-ADPR and ADPR. Of particular note, Juan and her team identified, in addition to the long-known ligand binding site in the C-terminal NudT9 homology domain, a second ligand binding site in the melastatin homology regions 1 and (MHR1/2). While the NudT9 homology domain served as an ADPR consuming enzyme in invertebrates, in vertebrates the enzymatic activity was lost, while binding of 2’deoxy-ADPR or ADPR is still necessary for channel opening.

## Poly(ADP-ribosyl)ation and PARPs

The second session on Tuesday afternoon, June 15, 2021, focused on poly(ADP-ribosyl)ation and PARPs and was chaired by Lee Kraus (Dallas, USA).

In the keynote talk Daniela Corda (Rome, Italy) discussed the PARPs with Mono-ADP-Ribosyl-Transferase activity, focusing on the identification of specific substrates to elucidate their cellular functions. The focus was on PARP12, that contributes to cell responses to stress by reversibly translocating from the Golgi complex to the stress granules, an event that causes the inhibition of the anterograde membrane traffic [7]. This has led to the finding that PARP12 regulates specific membrane traffic steps, by Mono-ADP-Ribosylating traffic controlling proteins such as Golgin-97 and members of the Rab family. Thus, the PARP12-dependent modification of Golgin-97 controls the export of E-cadherin at the Golgi complex by directing it to specific carriers destined to the basolateral membranes, where E-cadherin contributes to the maintenance of cell polarity and cell-cell junctions (Corda et al., in revision).

In the first short talk, Sunil Sundalam (Portland, USA) presented novel chemical proteomics approaches for profiling the NAD interactome. To identify novel NAD binding/interacting proteins, photo-affinity labeling probes, 2- and 6-ad-BAD, were developed. Known, e.g. PARP-1 and PARP-10, and unknown NAD/NADH-binding proteins were labeled in UV-dependent manner and identified by mass spectrometry [8].

Alexander Green (Ottawa, Canada) gave the second short talk and asked whether PARP1 would positively regulate muscle function and mass. Using data obtained from clinical trials with PARP-1 inhibitors and PARP-1-KO mouse model specific for mature muscle, PARP1 was found to preserve muscle mass while PARP inhibitors impair muscle mass maintenance.

Aravinthkumar Jayabalan (Johns Hopkins University, USA) in short talk #3 presented novel results showing that RNA viruses, including alphaviruses and coronaviruses, express a conserved macrodomain harbouring ADP-ribosylhydrolase activity to remove ADP-ribose moieties from target proteins. Of interest, the macrodomain is critical for viral replication and virulence. The underlying mechanism is the disassembly of cellular stress granules by the macrodomain’s ADP-ribosylhydrolase activity and releases translation factors to promote viral protein synthesis. The data indicate that viral macrodomains may become novel therapeutic targets for anti-viral strategies [9].

Mechanistic insights into degradation of the poly-ADP-ribosyl modification on proteins was provided by Johannes Rack (Oxford, UK). Utilizing homogenous, synthetic linear and branched PAR molecules showed that PARG is the main hydrolase of both PAR forms, while ARH3 can only degrade the linear one. Furthermore, detailing ARH3’s catalytic mechanism, involving substrate assisted rearrangement of a catalytic glutamate residue and induction of a new Mg^2+^ coordination geometry, revealed why the terminal serine-ADP-ribosyl modification is cleaved efficiently whereas linear PAR is a comparatively poor substrate [10, 11].

In the early career keynote Ivan Matic (Cologne, Germany) presented a phospho-guided chemical biology approach based on the Ser-ADP-ribosylation writer complex HPF1/PARP1 for generating precisely ADP-ribosylated peptides [12]. Integration of this methodology with phage display technology enabled the development of the first site-specific as well as broad-specificity antibodies to mono-ADP-ribosylation. Using these novel antibodies mono-ADP-ribosylation was found to be prevalent upon DNA damage. Poly(ADP-ribose) glycohydrolase (PARG) generates mono-ADP-ribosylated histones and PARP1 by rapidly degrading poly-ADP-ribosylation.

## Sirtuin biology

The first session on Wednesday morning, June 15, 2021, focused on Sirtuin biology was chaired by Vera Gorbunova (University of Rochester, USA). Sirtuins are a family of enzymes involved in the regulation of multiple biological processes such as gene silencing and transcription, DNA repair and metabolism all of which are crucial for aging and disease. Sirtuins use NAD+ as a substrate for deacetylation and mono-ADP-ribosylation reactions.

In first keynote talk, David Sinclair (Harvard Medical School) presented the model of epigenetic drift as a main cause of aging. DNA double strand breaks resulting from exogenous and endogenous stressors slowly erode the epigenetic chromatin packaging over the organism’s lifetime. DNA breaks and other forms of cellular injury such as nerve crush are attended to by epigenetic modifiers and, each time, the localization of these factors and epigenetic marks is not fully reset. Over time, this incomplete chromatin restoration results in changes in gene expression, a loss of cellular identity and function, and diseases we associate with aging, a process he calls “epigenetic drift” and cellular “exdifferentiation”.

Increasing the frequency of non-mutagenic DNA breaks in mice for three weeks results in accelerated aging, providing evidence for cause and effect. Epigenetic enzymes such as Sirtuins 1 and 6 maintain epigenomic stability, but their activity and abundance decline over time. One way to counteract this decline is to rejuvenate the epigenome by expressing reprogramming factors, such as Oct4, Sox2, and Klf4 (OSK), which reverses the DNA methylation age and youthful gene expression of neurons and restores the vision of old mice and mice with glaucoma, in a manner that requires the active, catalytic removal of DNA methylation via Tet1 and Tet2 [13].

In the first short talk, Daniel Levine (Northwestern University, USA) discussed the role of NAD+ signaling in circadian regulation. Elevation of NADH, which occurs during the morning with calorie-restricted diet in rodents, leads to inhibition of SIRT1 and its downstream metabolic genes. NADH signaling through SIRT1 couples epigenetic state with the nutrient restriction facing nocturnal animals at sunrise each day and opens a potential avenue for intermittent redox manipulation as a calorie-restriction mimetic [14].

In the second short talk, Matthew Simon (University of Rochester, USA) presented a novel gain of function SIRT6 mutation enriched in human centenarians. The mutation enhances SIRT6 mono-ADP ribosylation activity and leads to enhanced function of SIRT6 in DNA repair, suppression of transposable elements and killing of cancer cells. Surprisingly, the centenarian mutation reduces SIRT6 deacetylation activity arguing that mono-ADP-ribosylation function of SIRT6 is more important for its role in longevity. These results present the first evidence that the function of SIRT6 is linked to exceptional longevity in human.

Jonathan Gigas (University of Rochester, USA), in the third short talk, presented novel mouse models with altered JNK phosphorylation site on SIRT6. The mutation shifts the balance between SIRT6 bund to repetitive elements in heterochromatin and SIRT6 present on double strand breaks. SIRT6 localized to repetitive elements leads to lifespan extension, while SIRT6 biased towards DNA breaks results in elevated resistance to DNA damage without lifespan extension.

In the last short talk of the session, Andrey Nikiforov (Institute of Cytology, Russia) presented new data that purine nucleoside phosphorylase controls nicotinamide riboside metabolism in mammalian cells. After being imported into cells NR is intensively metabolized generating nicotinamide. Overexpression of purine nucleoside phosphorylase increased, while inhibition blocked NR conversion to nicotinamide *in vivo* and in cultured cells. Suppression of purine nucleoside phosphorylase potentiated the NAD synthesis from NR in human cells.

The junior keynote presentation delivered by Debra Toiber (Ben Gurion University of the Negev) focused on the roles of SIRT6 in DNA repair, DNA damage signaling and Alzheimer's disease. SIRT6 directly recognizes DNA damage through a tunnel-like structure that has a high affinity for DSB. SIRT6 relocates to sites of damage independently of signaling and known sensors. It activates downstream signaling for DSB repair by triggering ATM recruitment, H2AX phosphorylation, and the activation of both HR and NHEJ [15]. Additionally, SIRT6 has a novel role in preventing Alzheimer's disease by regulating the phosphorylation of Tau [16] and Tau-acetylation at K174. These results suggest that increased Tau-K174ac in AD case subjects results from DNA damage signaling and SIRT6 depletion [17]. Last, they showed a signature of pathological changes in gene expression in the brain with aging that are co-regulated by SIRT6 and YY1 [18].

## Metabolism and interventions

The second session on Wednesday afternoon, June 15, 2021, focused on effects of NAD+ on metabolism and NAD supplementation, was chaired by Raul Mostoslavsky (Harvard Medical School, USA). NAD+ intermediates such as NMN and NR have become widely advertised for their health promoting effects. Restoration of NAD^+^ concentrations in old or diseased mice through administration of NMN improves health; however, many questions regarding its efficacy in human and the biological mechanisms of action remain to be answered.

Shin-Ichiro Imai (Washington University, USA) gave the first keynote talk in the metabolism and interventions session. He presented new results from the first randomized, placebo-controlled, double-blind clinical trial to evaluate the effect of NMN on metabolic function in prediabetic postmenopausal women who were overweight or obese. NMN supplementation increased muscle insulin sensitivity and insulin signaling and promoted muscle remodeling. These results open a new avenue to develop NMN as a potential anti-aging intervention to manage diabetes and other age-related metabolic conditions.

In the first short talk Iva Guberovic from the group of Marcus Buschbeck (Carreras Research Institute, Spain) presented new analysis of the evolution of histone-mediated compartmental regulation of NAD metabolism. The macrodomain-containing histone variant macroH2A1.1 limits nuclear NAD+ consumption by inhibiting PARP1 in myotubes [19]. Analysis of the evolution of macroH2A showed that it originated in premetazoan protists where the metabolic function of macroH2A was associated with non-proliferative stages.

Karthikeyani Chellappa (University of Pennsylvania, USA) presented the second short talk on fate of the NAD precursors cycle in the gut. NAD is a redox cofactor essential to all living organisms, including microbes in the gut. Surprisingly, major proportion of NAD precursors for gut microbes are not derived directly from food, rather nicotinamide originating in the host circulation enters the gut lumen and serves as a precursor for microbial NAD synthesis. Concomitantly, host tissues utilize the nicotinic acid generated by microbes for NAD synthesis. These result show that, NAD precursors cycle between the host and gut microbiome to maintain NAD homeostasis.

In the short talk#3, Nora Kory (Harvard University, USA) presented identification of the long sought metazoan mitochondrial NAD transporter MCART/<soft-enter/>SLC25A51. MCART1-null cells showed large decreases in mitochondrial levels of NAD+ and NADH, while isolated mitochondria from cells overexpressing MCART1 showed greatly decreased or increased NAD uptake. Moreover, MCART1 functionally complemented a yeast mitochondrial NAD+ transporter. Thus, MCART1 provides a new target to manipulate mitochondrial NAD levels and to investigate the role of the mitochondrial NAD in physiology and disease [20].

Sarmistha Mukherjee (University of Pennsylvania) in the short talk #4 presented data that NR enhances liver regeneration after partial hepatectomy in SIRT3 or SIRT1-independent manner. Thus, providing supplemental NAD precursors may be effective strategies to promote recovery from liver injury [21].

The final early career keynote talk was delivered by Xiaolu Cambronne (University of Texas at Austin, USA). She presented collaborative work with Joseph Baur’s group (University of Pennsylvania, USA) that established MCART1/SLC25A51 as a major mammalian mitochondrial NAD^+^ transporter required for oxidative cellular respiration and that showed that it was sufficient for NAD^+^ uptake in a recombinant system [22]. She further presented models for selective ligand binding and regulation of how the opening of the carrier might be controlled.

The landmark papers—presented by Nora Kory and Xiaolu Cambronne—were published in Science Advances and Nature respectively. Notably, the function of SLC25A51 was further supported by a third publication from the Superti-Furga lab [23]. With the identification of SLC25A51/MCART1 as the major mitochondrial NAD^+^ carrier in mammals, a long-standing mystery in NAD^+^ biology has been resolved.

A highlight of the conference was “Meet the Editors” session featuring Rosalind Mott (Cell Metabolism) and Christoph Schmitt (Nature Metabolism). The editors answered questions and gave advice on topics from how to publish an impactful paper to what a career as a scientific editor is like. The session benefited both senior and junior researchers in the field. The conference concluded with a social hour at “wonder me” virtual bar.
